# Bespoke Drug Discovery Training for Low-Middle Income Countries

**DOI:** 10.1021/acsmedchemlett.3c00215

**Published:** 2023-07-12

**Authors:** Lauren A. Webster, Susan J. Farrell, Ian H. Gilbert, Kevin D. Read

**Affiliations:** Drug Discovery Unit, Wellcome Centre for Anti-Infectives Research, School of Life Sciences, University of Dundee, Dundee DD1 5EH, U.K.

**Keywords:** Drug Discovery, Capability, Medicinal Chemistry, Pharmacology

## Abstract

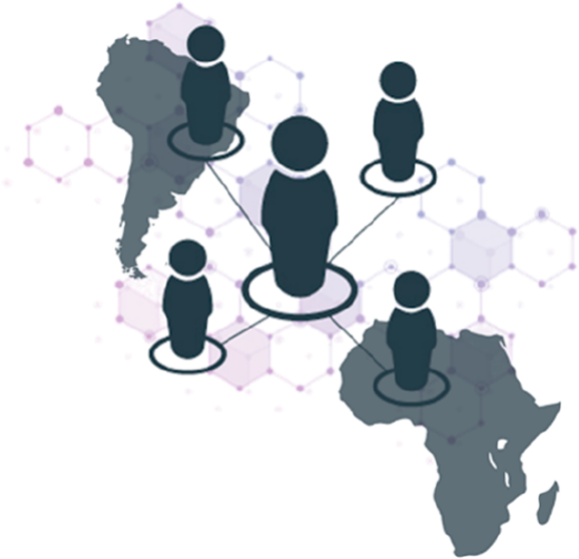

Working in drug discovery is difficult for many institutions due to the need for resources, funding, and in-country expertise. The Wellcome Centre for Anti-Infective Research (WCAIR) is responding to the unmet training needs for individuals/institutions working in drug discovery in low-middle income countries. Through their training program, individuals can undertake a practical placement, either online or at the center, with access to a dedicated trainer from their field of research. Practical placements are tailored to the needs of the individual/institute to enable capability building on return to their home institute. In addition to training placements, the center is focused on building partnerships by supporting institutes to work in drug discovery. Here we highlight WCAIR’s training program and the partnerships that have developed from this.

Drug discovery is a complex, resource-heavy and collaborative discipline. These traits make it incredibly difficult for academic institutions, particularly in developing nations, to work in this field.^1^ To strengthen in-country knowledge, the Wellcome Centre for Anti-Infective Research (WCAIR) delivers training for researchers working in neglected infectious disease space from low- and middle-income countries (LMICs).

WCAIR is located within the University of Dundee, Scotland (UK). The center encompasses the Drug Discovery Unit (DDU), Mode of Action, and parasitology, with a primary focus on neglected tropical disease (NTD) drug discovery research. Together, the specialties housed at WCAIR are medicinal chemistry, computational chemistry, screening biology, mode of action, structural biology, and drug metabolism pharmacokinetic (DMPK) teams—thus, offering a wide range of disciplines to undertake small-molecule drug discovery research for parasitic neglected infectious diseases. The DDU’s contribution to drug discovery has led to several clinical candidates in this disease area.^2,3^

This paper highlights the challenges and successes of the WCAIR training program and its continued involvement in supporting drug discovery in LMICs.

Since starting the training program in 2018, WCAIR has supported 48 trainees from 15 countries (Table 1). WCAIR accepts applicants from late-stage Ph.D. students through staff level and up to professorial level. Suitable trainees are those who have the means to disseminate their newly acquired knowledge and skills to peers and students. Added to this, it is important the trainee has full support from their group and institute to attend a placement, as trainees require a period of absence from their institute and everyday duties when they attend a placement at WCAIR.

**Table 1:** Geographic Reach of WCAIR Training Program Placements from 2018 to 2023

	Dundee Placement Total
**Africa**	**21**
Cameroon	2
Ghana	10
Malawi	2
Nigeria	4
South Africa	3

**Asia**	**1**
Malaysia	1

**Europe**	**4**
Belgium	1
Italy	1
Spain	1
UK	1

**North America**	**5**
Columbia	1
Cuba	3
USA	1

**South America**	**17**
Brazil	16
Uruguay	1

**Grand Total**	**48**

The WCAIR training program allows trainees from LMICs to focus on medicinal chemistry and DMPK with two dedicated trainers. Fully funded, each trainee receives a unique placement tailored to their own skills and infrastructure at their own institution. A placement consists of 3 months of online training followed by a 3–9-month practical placement, either at WCAIR or at their home institution via online tuition. The trainee assists with the design of their placement to ensure their personal and institutional needs are met. It is important to establish a relationship with their supervisor/line manager to facilitate relations and support the trainees’ newly acquired knowledge and skills upon returning to their home institution.

Drug discovery is a resource-heavy discipline, and as a result, academic institutions struggle to find the equipment necessarily to effectively support a drug discovery program. We have found all our trainees have resource issues. To help circumvent this, pre-arrival, our dedicated trainers spend time readjusting their systems to ensure the trainee will be able to perform the experiment when returning to their home institution. We investigate not only alternative methods but also alternative reagents, which the trainees will be able to acquire. We believe this step is vital for capability building in LMICs.

During a placement, trainees have full access to a dedicated trainer from their field of interest. Along with learning new skills and techniques, trainees are embedded into WCAIR’s drug discovery projects to expose them to the operations of a drug discovery team.^4−7^ Drug discovery is highly collaborative, and it is important that the trainee learns how to share and present data to members of the group, to interpret the different data and understand how decisions are made in the project matrix environment. This allows for open discussion and suggestions to drive the project forward to reach its goal. We have found some trainees are not aware of how to share information within their own area of expertise or with other specialties, and there is often a fear that their idea may be “stolen”. It is important to build this level of confidence and security in them to participate in drug discovery. WCAIR is fortunate it has many staff, all from multiple areas of research. Many of our trainees work in smaller teams back home and in some instances, no access to other discipline experts. Working in WCAIR teams, trainees are exposed to multiple projects and the discussions that go along with them. This further broadens their knowledge and understanding and enables them to implement it into their work more easily.

Until 2021, WCAIR also offered placements in biology, with a focus on assay development for screening. However, trainees struggled to implement this learning on their return, often due to limited access to compound collections on top of the issues surrounding purchasing of consumables that all trainees seem to face. We made the decision to focus on chemistry and DMPK placements for now, with a view to reintroducing biology-related placements in the future. In the interim we intend to ensure that individuals can gain knowledge from short courses or freely available online content.

On their return, trainees develop their own research and disseminate their knowledge to peers, staff, and students at their home institutions. Training provides a good start to capability building; however, many obstacles still exist for researchers in LMICs. With that in mind, WCAIR has been developing partnerships with trainees and institutions to provide support with grant applications and training. Bringing researchers together to share experience and resources will be invaluable for any institutes working in drug discovery.

Using Ghana as an example, several small pilot projects were funded through the Global Challenges Research Fund (GCRF) in natural product and cryptosporidiosis research (unpublished). Further funding from the Academy of Medical Sciences (AMS) allowed WCAIR to support the Ghanaian researchers to develop a network in drug discovery and a series of working groups to identify challenges and training needs. These groups have identified a number of training needs where WCAIR can assist, including analytical and medicinal chemistry training. Added to this, additional organizations were sought to support in other areas where WCAIR could not. WCAIR was a recipient of the MSD Richard T Clarke Fellowship that utilized the experience of fellows from an industrial setting to provide training to support the development of the drug discovery platform within Ghana. Training was successfully delivered in three areas: DMPK, oncology, and clinical trials through online platforms.

In addition, WCAIR is a partner in a recent Bill & Melinda Gates Foundation award to the University of Ghana, which is a both a malaria drug discovery program and a capability building award. This award will fund the first fully integrated drug discovery program to run in the country. This includes setting up of the first series of DMPK assays in the country. Many of the individuals involved in that award have attended training through WCAIR and H3D (University of Cape Town, South Africa) and will be completing additional training placements at either institution.

The WCAIR training program has gained insight into the strengths and gaps for researchers in LMICs tackling drug discovery. Our main areas of focus have been South America and Africa; however, we are open to support scientists in any low- and middle-income country.

Through interactions with trainees and collaborators, we have seen differences between capabilities vary widely between countries and continents. For example, countries in South America tend to have more established chemistry research, and as such have more experience in this field compared to many countries in Africa. They tend to have access to more equipment, consumables, and shared facilities and have established programs in both natural product and synthetic chemical libraries to initiate drug discovery. This in turn opens up more opportunities for research and collaboration.

WCAIR trainees have learned to adapt our methodologies to laboratories that have limited infrastructure, enabling us to support more trainees and institutes. This has been evident when assisting with the establishment of DMPK in Ghana. The WCAIR training program continues to be a success, with places already filling up for 2024. WCAIR has assisted in unlocking the drug discovery potential of LMICs. Together with our WCAIR training team, funders, collaborators, and trainees, we will continue to support capability development in drug discovery for researchers in LMICs. Although our main focus is around NTDs, many of the areas we support would be transferable to researchers working on other disease areas.

## Abbreviations

WCAIR: Wellcome Centre for Anti-Infective Research
LMIC: Low-Middle Income Country
GCRF: Global Challenges Research Fund
AMS: Academy of Medical Sciences
