# Correction to “Bespoke Drug Discovery Training for Low-Middle Income Countries”

**DOI:** 10.1021/acsmedchemlett.3c00399

**Published:** 2023-10-05

**Authors:** Lauren A. Webster, Susan J. Farrell, Ian H. Gilbert, Paul G. Wyatt, Kevin D. Read

Pages 1014 and 1016. The list of authors has been updated to include Paul G. Wyatt, whose information is as follows:

**Paul G. Wyatt** – *Drug Discovery Unit, Wellcome Centre for Anti-Infectives Research, School of Life Sciences, University of Dundee, Dundee DD1 5EH, U.K.*; orcid.org/0000-0002-0397-245X

